# Late Lung Metastasis in a Patient with a Clear Cell Chondrosarcoma: An Indication for a Life-Long Follow-Up?

**DOI:** 10.1155/2021/7205649

**Published:** 2021-12-02

**Authors:** Paulien West, Celine Jacobs, Michael Saerens, David Creytens, Gwen Sys, Lore Lapeire

**Affiliations:** ^1^Faculty of Medicine and Health Sciences, Ghent University, Ghent, Belgium; ^2^Department of Medical Oncology, Ghent University Hospital, Ghent, Belgium; ^3^Department of Pathology, Ghent University Hospital, Ghent, Belgium; ^4^Cancer Research Institute Ghent (CRIG), Ghent University, Ghent, Belgium; ^5^Department of Orthopaedics and Traumatology, Ghent University Hospital, Ghent, Belgium

## Abstract

**Background:**

Clear cell chondrosarcoma (CCCS) is a rare subtype of chondrosarcoma and comprises between 1.6% and 2.5% of all chondrosarcoma. They are known to be chemo- and radiotherapy resistant; surgical resection is therefore the therapy of choice.

**Methods:**

We present a 63-year-old woman with a progressive lung nodule 20 years after initial diagnosis and treatment of a clear cell chondrosarcoma of the right os naviculare.

**Results:**

On serial CT scans of the chest, an asymptomatic, slowly growing nodule in the left upper lung lobe was detected. CT-guided transthoracic biopsy of this nodule confirmed the diagnosis of a chondrosarcoma lung metastasis. Video-assisted thoracoscopic wedge resection was performed with complete removal of the nodule. The patient recovered well from surgery and remains in good health during further follow-up.

**Conclusion:**

Given the tendency of clear cell chondrosarcoma to recur and metastasize after extended periods of time, a long-term, possibly life-long follow-up and clinical surveillance is advisable in these patients.

## 1. Introduction

Clear cell chondrosarcoma (CCCS) is a rare subtype of chondrosarcoma and comprises between 1.6% and 2.5% of all chondrosarcoma [1]. Initial symptoms of CCCS typically start 1.5 years before diagnosis and are often indolent, which is characteristic for the slow progression of CCCS [1–3]. CCCS and chondroblastoma share common radiographic features, but distinct histopathological features [1, 4]. Wide resection is the mainstay of treatment as the disease is resistant to chemo- and radiotherapy and tends to recur after incomplete resection [1, 5, 6]. Rarely, late recurrences and even distant metastases may occur [7]. We present a case of a 63-year-old female with a history of CCCS of the right os naviculare, who developed a lung metastasis 20 years after resection.

## 2. Case Presentation

**Figure 1:**
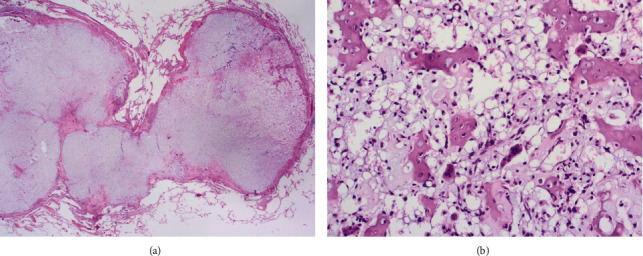
Histopathological image of the primary tumor. Atypical rounded cells with abundant clear cytoplasm, arranged in lobules, and admixed with trabeculae of woven bone and osteoclast-like giant cells (hematoxylin and eosin, original magnification (a) 100x and (b) 200x).

In February 1996, a 40-year-old Caucasian woman consulted an orthopaedic surgeon because of persistent pain in the right foot. On radiograms, a lytic tumoral process was discovered in the right os naviculare. Curettage was performed, and anatomopathological revision of the obtained tissue at the Mayo Clinic Rochester (Dr. Unni) confirmed the diagnosis of a clear cell chondrosarcoma (Figure 1). In June 1996, local progression of the disease was discovered and an amputation of the right lower leg was performed. Subsequently, she was followed with annual chest X-ray examination and clinical examination by her treating orthopaedic surgeon.

**Figure 2:**
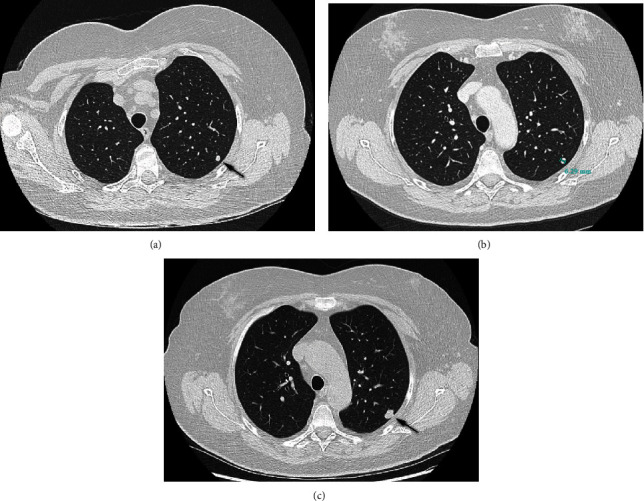
Radiographic images of the suspected metastases. CT scan of a dense opacity in the left upper lung lobe of 6 mm in April 2016 (a). The nodule was unchanged after 6 months of follow-up (b). In December 2019, the nodule increased to 13.5 mm (c).

In April 2016, her chest radiogram showed a new dense opacity in the left upper lung lobe. On CT scan, two opacities in the left upper lobe were detected, with a diameter of 6 mm and 3 mm, respectively (Figure 2). After multidisciplinary consultation, close follow-up was advised given the aspecific and small diameter of the lesions. Three months later, only one lesion remained on CT scan. Further follow-up with annual radiograms was organised. In December 2019, a slight increase in diameter in the nodule in the left upper lobe was seen. Subsequently, a new CT scan was performed, which showed a single nodule of 13.5 mm. Apart from a dry cough, the patient was asymptomatic at the time of this finding. A lung function examination was performed and showed no abnormalities. Further staging with an abdominal CT showed no other lesions suspected for metastases. There was no history of smoking. A CT-guided lung biopsy was performed, and the patient was referred to our tertiary sarcoma centre for further diagnostic examination and treatment because of the probable diagnosis of a chondrosarcoma metastasis.

At the time of referral, the patient was in good health and had no symptoms other than a transient dry cough. She did not have complaints of weight loss or exhaustion. Clinical examination showed no abnormalities besides the lower right leg amputation after surgery for the chondrosarcoma.

**Figure 3:**
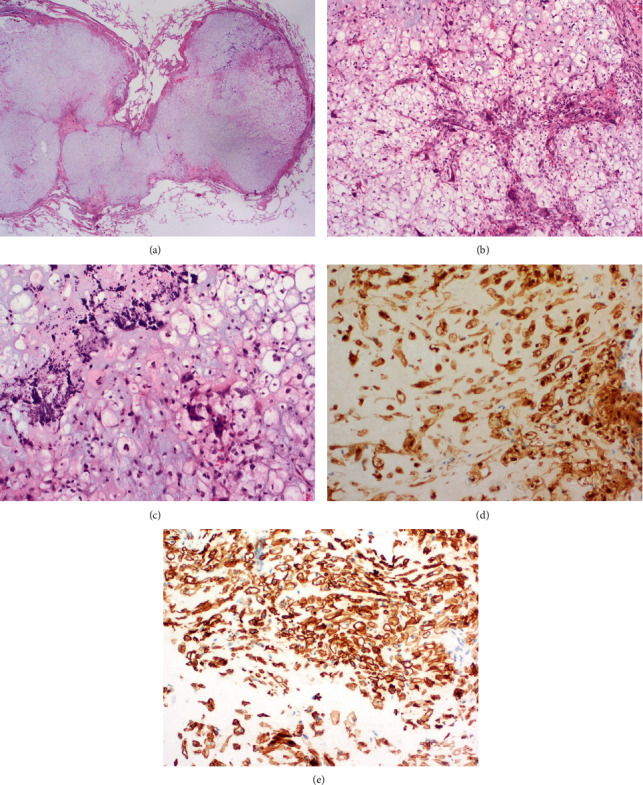
Lung metastasis. Histology of the wedge resection of the left upper lung lobe showing a 15 mm large, well-circumscribed chondroid tumoral lesion (hematoxylin and eosin, original magnification 12x (a)). Atypical rounded cells with a centrally placed, large round nucleus and abundant clear cytoplasm (hematoxylin and eosin, original magnification 100x (b)). Presence of osteoclast-like giant cells, a chondroid matrix, and calcifications (hematoxylin and eosin, original magnification 200x (c)). Diffuse expression of the tumor cells for S100 (original magnification 200x (d)) and Podoplanin (D2-40) (original magnification 200x (e)).

Histopathological examination of the lung biopsy showed the presence of an atypical chondroid tumoral proliferation composed of atypical spindled to rounded chondroid cells embedded in a chondroid matrix (Figure 3). No obvious clear cell morphology, high-grade cytonuclear atypia, or mitotic activity was observed in this biopsy. Immunohistochemically, the atypical cells showed a diffuse expression of S100 and Podoplanin (D2-40). These morphological and immunohistochemical findings were suspected for a metastasis of a chondrosarcoma.

After discussion in our multidisciplinary bone and soft tissue sarcoma board, a video-assisted thoracoscopic wedge resection was performed. Histopathological examination of the resected lung nodule showed a 15 mm large, well-circumscribed chondroid tumoral proliferation composed of atypical rounded cells with a centrally placed, large round nucleus and abundant clear to slightly eosinophilic cytoplasm. Intermingled within these atypical clear cells, osteoclast-like giant cells, a chondroid matrix, and calcifications were observed. The atypical cells stained strongly with antibodies against S100 and Podoplanin (D2-40), further confirming the diagnosis of a lung metastasis of the known clear cell chondrosarcoma (Figure 3).

She recovered well from surgery. Given the extended time frame between the primary tumor and the development of this single lung metastasis (20 years) and the insensitivity of clear cell chondrosarcomas to chemotherapy, a conservative approach was held with regular imaging as follow-up, meaning a chest CT every six months during the first year.

## 3. Discussion

Clear cell chondrosarcoma (CCCS) is a rare subtype of chondrosarcoma and comprises between 1.6% and 2.5% of all chondrosarcoma [1]. Unni et al. were the first to describe these tumors in 1976 (2). CCCS usually involves the proximal part of the femur or humerus and is more present in men than in women [5, 8]. These tumors are most commonly seen in the third through fifth decade of life [1].

Initial symptoms of CCCS typically start 1.5 years before diagnosis and are often indolent, which is characteristic for the slow progression of CCCS. Pain in the affected bone is the typical presenting symptom in 50% of the patients. Sometimes, patients with a CCCS present with a pathological fracture [1–3].

Imaging shows a lytic lesion with a characteristic peripheral border of sclerosis and associated matrix calcification which can also be seen in a chondroblastoma. CCCS and chondroblastoma can be difficult to distinguish radiologically. Differential diagnosis is based upon age; chondroblastoma usually occurs in children or adolescents, whereas CCCS occurs rather in the third to fifth decades of life [1].

Histology is important to make the definitive diagnosis. CCCS is characterized by rounded or polygonal cells with abundant pale, clear, to slightly eosinophilic cytoplasm, resembling hypertrophic cells of the growth plate. The tumor cells are arranged in lobules and sheets and are frequently admixed with trabeculae of woven bone and osteoclast-like giant cells (Figure 1). Immunohistochemically, the tumor cells are strongly positive for S100. The histopathological differential diagnosis includes metastatic clear cell renal cell carcinoma, chondroblastoma, and osteosarcoma, as well as (depending on localisation) a notochordal tumor [4].

Clear cell chondrosarcomas are commonly described to be a low-grade malignancy and are known to be resistant to chemo- and radiotherapy. Surgical resection is therefore the treatment of choice in these tumors. Because of the high rate of local recurrence after curettage or excision (up to 86%), a resection of the tumor with wide margins is advised [1, 3, 6]. As seen in our patient, an initial curettage was insufficient and amputation of the right lower leg was necessary after local progression four months after the initial surgery.

Given the restricted number of formal prospective studies, strict rules about follow-up are absent. Follow-up according to the ESMO bone sarcoma guidelines should include a physical examination of the tumor site, local imaging, and chest X-ray/CT scan every 3 months for the first 2 years, every 6 months for years 3-5, every 6-12 months for years 5-10, and thereafter every 6-24 months according to local practice and other factors [9]. Late metastasis, as seen in our case, may occur more than 10 years after the initial diagnosis and there is no universally accepted stopping point for tumor surveillance [9].

Generally, the risk of local recurrence and metastatic disease from CCCS is considered low [7]. Most local recurrences occur in the first 5 years; however, case reports have been published describing local recurrences later than 10 years after initial resection in 15% of cases (Table 1) [3, 7]. This emphasizes the need for a thorough and long-term follow-up of patients with CCCS [7].

As previously described, adequate surgical margins have a crucial impact on the survival of patients with CCCS. Patients with a clear resection margin at the time of initial surgery showed no local recurrence during follow-up and longer disease-free survival than patients with marginal or intralesional resection [3, 7, 10].

CCCS has a metastatic potential in the lung, bone (mostly spine), and rarely brain tissue [1, 2, 7, 11]. It is known to metastasize relatively late, and almost 25% of metastatic disease is detected more than 10 years after surgery [3, 7, 10, 12]. The prevalence of local recurrence and the grading of the tumor are prognostic factors of metastatic disease [7]. This supports the choice for aggressive surgical treatment in local recurrence [3, 11], exemplified by our case.

Multiple case reports (Itälä et al., Donati et al., and Laitinen et al.), including ours, show that the development of metastasis in CCCS seems to be slow and multiple resections can be done with a survival of many years or even decades (Table 1). According to international guidelines, surveillance should be at least 10 years and perhaps in this particular tumor type even lifelong due to the tendency of this tumor to slowly progress over time [7, 9–11]. However, this needs to be weighed against the economic and psychological burden of this extensive follow-up on a case-by-case basis.

When comparing the existing literature with our case, it is clear that when intralesional surgery is performed, a higher risk of local recurrence and metastatic disease is created. Therefore, when a lytic bone lesion is seen on imaging, a malignancy should always be kept in mind, and before surgery is performed, the case should be discussed in an expert centre with expertise in bone and soft tissue malignancies to maximize the prognosis of the patient.

## 4. Conclusion

Although most local recurrences of CCCS are observed during the first five years, CCCS may have a tendency to recur and metastasize after extended periods of time. We illustrated this in a clinical case. A long-term, perhaps lifelong, follow-up and clinical surveillance including physical examination of the tumor site, local imaging, and chest X-ray/CT scan are advised in these patients. Given the slow course of the disease and the insensitivity to radio- and chemotherapy, local recurrences and metastases should be treated with surgical resection wherever possible to maximize the overall survival of the patients.

## Figures and Tables

**Table 1:** Data of case reports describing patients with CCCS and local recurrence or metastatic disease.

Author	Patients (*n*)	Publication	Location of diagnosis	Type of resection	Local recurrence (*n*; %)	Time of local recurrence	Metastatic disease (*n*; %)	Location of metastasis	Time of metastasis (range)	Outcome
Unni et al. [2]	16	1976	N/A	1 X N/A 8 X R0 7 X R1/2	4; 25%	0.25 y–5 y (median 2 y)	6; 38%	5 X lung 2 X bones	6 y–19 y (median 6.7 y)	1 X N/A 10 X NED 1 X AWD 4 X DOD

Bjornsson et al. [7]	47	1985	N/A	5 X N/A 23 X R0 19 X R1	16; 34%	4.6 y	7; 15%	5 X lung 3 X bone	3.5 y–7 y (median 4 y)	9 X N/A 22 X NED 8 X AWD 8 X DOD

Komiya et al. [13]	1	1986	Femur	R2	0	—	1	Bones/lung	4 y	DOD

Lee et al. [14]	1	1989	Femur	R1	1	5.3 y	0	—	—	NED

Bagley et al. [12]	2	1993	N/A	R0	0	—	—	1; 50%	23 y	1 X NED 1 X AWD

Ron et al. [15]	1	1995	Rib	R0	1	0.4 y	1	Lung	1 y	DOD

Ayoub et al. [16]	6	1999	Femur, ilium	3 X R0 3 X R1/2	3; 50%	2.8 y–19 y (mean 5 y)	1; 17%	Lung	5.5 y	4 X NED 1 X AWD 1 X DOD

Hartwright et al. [17]	1	2000	Femur	R0	1	19 y	0	—	—	NED

Kalil et al. [4]	3	2000	Femur	2 X R0 1 X R1	2; 67%	0.6 y; 6 y	3, 100%	3 X lung 2 X bones	1.2 y–6 y (median 5 y)	3 X DOD

Itälä et al. [3]	16	2005	Femur, humerus, ulna, vertebra, rib, sternum	10 X R0 6 X R1	3; 19%	0.15–1.8 y (median 1.6 y)	3, 16%	1 X lung 2 X bones	4 y–16.3 y (median 7 y)	11 X NED 4 X AWD 1 X DOD

Kawano et al. [18]	1	2005	Left tibia	R0	0	—	1	Bone	4 y	NED

Srikanth et al. [19]	1	2005	Olecranon	R0	0	—	1	Lung	N/A	DOD

Simsek et al. [20]	1	2005	Femur	R2	1	3 y	0	—	—	NED

Donati et al. [10]	18	2008	Femur, tibia, humerus, astragalus bone, vertebral body, scapula, rib, pubic ramus, acetabulum	17 X R0 1 X R1	5; 28%	0.5–24 y (median 4.6 y)	2; 11%	Bone	1 y–5.3 y (mean 3.1 y)	15 NED 3 AWD

Elojeimy et al. [21]	1	2013	Humerus	R0	0	—	1	Bone	1 y	1 X AWD

Jiang et al. [22]	5	2014	Femur, vertebra, rib, sacrum	3 X R0 2 X R1	3; 60%	1 y–8 y (median 2.5 y)	3; 60%	Bone	9.1–20 y (median 10 y)	1 X NED 3 X AWD 1 X DOD

Laitinen et al. [11]	1	2014	Left proximal femur	R1	1	29 y	100%	Lung	29 y	AWD

Nagmani et al. [23]	1	2015	Calcaneum	R0	0	—	1	Lung	1.5 y	AWD

Moura et al. [24]	1	2016	Femur	R1	1	16 y	0	—	—	NED

Klein et al. (9)	7	2019	Proximal femur (5), proximal tibia (1), proximal humerus (1)	7 X R0	2; 30%	0.5 y	1; 14%	Lung	0.75 y	6 X NED 1 X DOD

Caetano de Oliveira et al. [25]	1	2021	Left proximal femur	R1/2	1	0.25 y	1	Rib	22 y	NED

Own case	1		Right os naviculare	R1/2	1	0.3 y	1	Lung	20 y	NED

NED: no evidence of disease; AWD: alive with disease; DOD: dead of disease.
